# Out-of-pocket payment for primary healthcare in the era of national health insurance: Evidence from northern Ghana

**DOI:** 10.1371/journal.pone.0221146

**Published:** 2019-08-20

**Authors:** Edmund Wedam Kanmiki, Ayaga A. Bawah, James F. Phillips, John Koku Awoonor-Williams, S. Patrick Kachur, Patrick O. Asuming, Caesar Agula, James Akazili

**Affiliations:** 1 Regional Institute for Population Studies, University of Ghana, Accra, Ghana; 2 Department of Population and Family Health, Mailman School of Public Health, Columbia University, New York, New York, United States of America; 3 Policy, Planning, Monitoring and Evaluation Division, Ghana Health Service, Accra, Ghana; 4 University of Ghana Business School, Legon, Accra, Ghana; 5 Navrongo Health Research Centre, Ghana Health Service, Navrongo, Upper East Region, Ghana; Ruprecht Karls University Heidelberg, GERMANY

## Abstract

**Background:**

Ghana introduced a national health insurance program in 2005 with the goal of removing user fees, popularly called “cash and carry”, along with their associated catastrophic and impoverishment effects on the population and ensuring access to equitable health care. However, after a decade of implementation, the impact of this program on user fees and out-of-pocket payment (OOP) is not properly documented. This paper contributes to understanding the impact of Ghana’s health insurance program on out-of-pocket healthcare payments and the factors associated with the level of out-of-pocket payments for primary healthcare in a predominantly rural region of Ghana.

**Methods:**

Using a five-year panel data of revenues accruing to public primary health facilities in seven districts, We employed mean comparison tests (t-test) to examine the trend in revenues accruing from out-of-pocket payments vis-à-vis health insurance claims for health services, medication, and obstetric care. Furthermore, generalized estimation equation regression models were used to assess the relationship between explanatory variables and the level of out-of-pocket payments and health insurance claims.

**Results:**

Out-of-pocket payment for health services and medications declined by 63% and 62% respectively between 2010 and 2014. Insurance claims however increased by 16% within the same period. There was statistically a significant mean reduction in out-of-pocket payment over the period. Factors significantly associated with out-of-pocket payments in a given district are the number of community health facilities, availability of a district hospital and the year of observation.

**Conclusion:**

The study provides evidence that Ghana’s national health insurance program is significantly contributing to a reduction in out-of-pocket payment for primary healthcare in public health facilities. Efforts should therefore be put in place to ensure the sustainability of this policy as a major pathway for achieving universal health coverage in Ghana.

## Introduction

The United Nations Sustainable Development Goals (SDGs) reinforce the need for national policies that ensure the financial risk protection of their citizens against the cost of unforeseen ill-health. Together with developing geographic access, financial access to essential care represents a major prerequisite for achieving Universal Health Coverage (UHC) [1]. Globally, it is estimated that over 150 million people face financial catastrophe while about 100 million are pushed into poverty each year due to direct out-of-pocket healthcare payments [2]. In addition, direct out-of-pocket healthcare payments have detrimental effects on the allocation of household disposable income for basic needs such as food, shelter, clothing, education and utilities among others [3, 4]. This creates the need for health systems to ensure financial protection of individuals and households against the economic burden of ill health.

Recognizing this, the World Health Assembly resolution (WHA) 58.33 called on member countries to commit to attaining UHC [5] by ensuring that all people have access to healthcare services when needed without financial impediments. It encompasses three dimensions: the proportion of people covered, the range of services covered and the proportion of health costs covered [2, 6].

Health insurance is often promoted as a means to achieve equitable financing of health care. By pooling risk and resources, health insurance has the potential to improve access to healthcare while providing risk protection against the cost of healthcare expenditure [7, 8]. Ghana was one of the few developing countries that took a bold policy to implement a national health insurance program. Ghana introduced the National Health Insurance Scheme (NHIS) in the year 2005 with the goal of removing financial barriers to accessing healthcare and protecting all citizens from catastrophic healthcare payments that arise from direct health care payment at the point of service delivery [9–12]. It is based on a contributory model where service benefits are restricted to contributors except people under the age of 18 years, pregnant women, pensioners, those above 65 years and indigents [9, 13, 14]. Enrolment unto the NHIS is mandatory by law. However, the fact that it is a social policy coupled with the large proportion of Ghanaians working outside formal employment settings, enforcement of its mandatory requirement is fraught with difficulties. Thus, the scheme relies on the voluntary registration of members from the informal sector [10].

The NHIS is financed by premium payments by subscribers, a 2.5% national health insurance levy on Value-added Tax (VAT) collected on selected goods and services, and another 2.5% deduction from workers’ contributions to the Social Security and National Insurance Trust (SSNIT) fund. Other sources of funding to the NHIS are government of Ghana budget allocations, grants, donations and proceeds of investments made by the national health insurance council [9, 15, 16]. The NHIS benefit package is said to cover about 95% of all disease conditions in Ghana; services covered by the scheme include outpatient services, essential drugs, inpatient accommodation, maternity care including cesarean delivery, dental care, and eye care among others [9].

Previous studies have shed light on various aspects of Ghana’s health insurance scheme, including helping to understand the determinants of enrolment [14, 15, 17], the perceptions and experience of stakeholders with the NHIS [9], its contribution to health facility utilization [18], its alignment with primary healthcare goals [12], how the informal sector could be more actively involved [10], and its financial protective effects [11, 19–22] among others. While there is population-level evidence that Ghana’s national health insurance is contributing to a reduction in catastrophic healthcare payments and indeed has financial protective capabilities [19–21], some studies also reveal that out-of-pocket health payment is still prevalent and pervasive in Ghana despite the presence of the scheme [21, 23]. For instance, the most recent demographic and health survey report reveals that one-third of women covered by the NHIS still made direct health care payments for medicines and services [23].

In the face of the mixed evidence on the impact of Ghana’s national health insurance program on direct out-of-pocket payments, there is the need for more empirical studies using different approaches to examine the extent to which Ghana’s national health insurance policy is achieving its goal of impacting on out-of-pocket healthcare payments and financial risk protection against the cost of unforeseen ill health. Moreover, evaluating the impact of health financing interventions is complex, and available evidence has been inconsistent [21, 22]. Health insurance schemes therefore require continuous monitoring and evaluation in order to inform policy and practice improvement. This study thus adopts a provider perspective by using health facility-based revenue data to assess the impact of Ghana’s national health insurance program on out-of-pocket health payments and contributes to the evidence-base of the financial risk protection provided by its implementation. More specifically, the study seeks to 1) examine the trends in out-of-pocket healthcare payments and health insurance claims, 2) test the significance of any changes in out-of-pocket healthcare payments and health insurance claims over a five-year period and 3) explore the factors associated with levels of out-of-pocket healthcare payments and insurance claims.

## Materials and methods

### Source of data and study setting

This paper used a five-year panel data collected from public primary healthcare facilities in seven districts of the Upper East Region (UER) of northern Ghana. The data was collected as part of the costing arm of a five-year health system strengthening and research program known as the Ghana Essential Health Intervention Project (GEHIP) that was implemented from 2010 to 2015. This data was collected to help assess the cost-effectiveness of the GEHIP project. (Details of GEHIP are described elsewhere) [24–28].

The UER is one of the ten administrative regions of Ghana. It ranks among the three poorest and most remote regions in the country with a population of over 1 million people [29] and is located in the northeastern corner of Ghana within the Savannah ecological belt. Subsistence agriculture is the predominant occupation of the indigenes, with only one short rainy season and deteriorating soil fertility. Poverty is pervasive, formal educational status is low and migration to the southern part of Ghana during the dry season is a common practice [25, 30, 31]. The seven districts from which data for this study were collected are Bolgatanga Municipality, Builsa District, Bongo District, Talensi-Nabdam District, Bawku Municipality, Bawku West District and Garu-Tempani District. These seven districts make up a total population of about 906,459 at the time of the study. As at 2014 when data collection was ongoing, five of the districts had district hospitals; also 33 health centers and a total of 106 community-based healthcare compounds were present within the study area.

### Data collection and analysis

Revenue data were systematically collected on all public primary healthcare facilities in the seven districts that were involved in the GEHIP project on a quarterly basis from the year 2010 to 2014. Quarterly aggregated data were collected by trained research assistants from the seven District Health Management Teams (DHMT) and entered into excel templates. Internally generated funds were extracted for separate analysis and data capture permitted separation of funds from national health insurance claims from funds derived from direct out-of-pocket patient payments. Data entries specified amounts in Ghanaian currency, but all data were transformed into US dollar equivalents specified by the Bank of Ghana for the midpoint of each study year. Disaggregation of the data was possible for three major categories of healthcare revenue, namely: medications, services, and obstetric care. For bivariate descriptive analysis, observation points were all 7 districts over a five year period; for regression analysis, data were disaggregated by quarter and by type of source of payment, out-of-pocket versus NHIS reimbursement related revenue the sample size was 140.

Descriptive statistics is first used to present out-of-pocket health payment and health insurance claims by medications, services and obstetric care for 2010–2014. The year 2010 was used as the base year for the purpose of comparisons. While 2010 represents about six years into the implementation of Ghana’s national health insurance policy, this is the initial point at which our data were collected.

Next, using STATA version 14 software, we perform a mean comparison test to determine and compare statistical differences by pairing out-of-pocket payments and insurance claims of 2010 (the baseline year) with each of the subsequent years (i.e. 2011, 2012, 2013 and 2014). We report standard errors and p-values of all paired cases.

Finally, we explored the relationship between revenues accruing from out-of-pocket payments and health insurance claims with merged data providing trends in five explanatory variables and differenced out-of-pocket payment versus insurance reimbursements. To adjust for autoregressive error that arises from repeated observation, we employ Liang and Zeger “Generalized Estimation Equation (GEE)” regression models [32]. The GEE approach permits estimation of relationships of factors influencing the level of out-of-pocket payment and insurance claims correcting effect parameters and standard errors for autoregressive related bias that could arise if district repeat observation effects were ignored [33, 34].

The independent variables used in this analysis were: year of observation, quarter of observation, population of district, number of community-based health facilities, number of health centers and the presence of a district hospital. These variables were selected because of their potential to influence the level of out-of-pocket healthcare payment and insurance claims. For instance, year of observation and quarter of observation are both time variables, they were included to test the effect of changes in time on the outcome variables. However, it was important to have both in the models because quarter of observation is subject to seasonal variation whereas year of observation is not. District population was included in the independent variables to test whether there exists any relationship between the outcome variables and population size of a district. The number of community-based facilities, number of health centers and the presence of a district hospital are all health provider points. These were included to examine if the number of these different patient care points in a district have any association with the level of out-of-pocket payment and insurance claims.

The basic assumption in this study is that a trend providing evidence of progressively higher insurance claims relative to corresponding trends in out-of-pocket payments, suggests that the NHIS program is plausibly reducing out-of-pocket payments and progressively offsetting financial barriers to healthcare, thereby contributing to Ghana’s health sector universal health coverage goal.

This basic assumption is associated with limitations. Using revenues as a proxy measure may not adequately address the tendency for insurance claims to increase due to repeated visits. This limitation notwithstanding, understanding the changes and patterns of revenue sources of health facilities in the phase of a national insurance program is important for organizational planning and operational policies. Before the introduction of the NHIS, almost all internally generated funds (revenues) of health facilities in Ghana were from out-of-pocket healthcare payments. However, with the introduction of the NHIS, revenues are now split between these two sources, a development that could provide a useful indicator for gauging NHIS performance. By examining the relative trends in these two revenue streams at a provider level, we also aim to reveal some of the economic considerations that may influence providers and district manager’s decisions about where to focus their effort, resources and personnel in ways that national policy makers may not have anticipated.

### Ethical consideration

This paper emanates from the Ghana Essential Health Interventions Project (GEHIP) which obtained ethical approval from the Ghana Health Service Institutional Review Committee as well as the Navrongo Health Research Center Ethics Review Board and the Columbia University Research and Compliance Administrative System prior to the conduct of the study. De-identification of data was done before analysis to ensure confidentiality and anonymity.

## Results

Table 1 shows the revenues from out-of-pocket payments and health insurance claims in the seven study districts over the 2010–2014 periods. It is observed that out-of-pocket payment for healthcare at the point of care reduced significantly over the period. For instance, out-of-pocket payment for medications reduced by 62% while that of services reduced by 63% in 2014 using the year 2010 as the base year.

**Table 1:** Revenues from out-of-pocket payments and insurance claims expressed in mid-year US $ equivalents (2010–2014).

**Out-of-Pocket payment**								
**Year**	**Medicines**		**Services**		**Obstetric care**		**Total**	
	**Amount**	**% Change**	**Amount**	**% Change**	**Amount**	**% Change**	**Amount**	**% Change**
**2010**	64,753.74	-	51,289.16	-	-	-	116,042.90	-
**2011**	57,712.67	-10.87	36,907.84	-28.04	-	-	94,620.51	-18.46
**2012**	45,590.81	-29.59	32,839.93	-35.97	-	-	78,430.75	-32.41
**2013**	42,121.98	-34.95	29,249.20	-42.97	-	-	71,371.18	-38.5
**2014**	24,936.51	-61.49	19,246.92	-62.47	-	-	44,183.43	-61.92
Total	**235,115.71**		**169,533.05**		-		**404,648.76**	
**Health Insurance Claims**								
**Year**	**Medicines**		**Services**		**Obstetric care**		**Total**	
	**Amount**	**% Change**	**Amount**	**% Change**	**Amount**	**% Change**	**Amount**	**% Change**
**2010**	1,050,963.54	-	600,384.49	-	157,721.86	-	1,809,069.88	-
**2011**	1,075,459.58	2.33	657,471.90	9.51	262,691.54	66.55	1,995,623.02	10.31
**2012**	1,162,036.26	10.57	742,906.70	23.74	229,201.18	45.32	2,134,144.14	17.97
**2013**	1,403,336.84	33.53	822,940.72	37.07	302,967.71	92.09	2,529,245.27	39.81
**2014**	1,145,589.36	9.00	677,536.16	12.85	276,593.39	75.37	2,099,718.90	16.07
Total	**5,837,385.58**		**3,501,239.97**		**1,229,175.67**		**10,567,801.22**	

2010 is used as the reference point for % change

Also, in all cases, there was an increase in insurance claims. For instance, claims for medications increased by 34% in 2013 and then 9% in 2014. Those for services increased by 37% in 2013 and then remained at 13% in 2014. Health insurance claims were observed to have taken the overall cost of obstetric care. From $157,721.86 in total insurance claims in the year 2010, this figure increased by 92% in 2013 and then settled at 75% of the 2010 baseline in 2014.

**Fig 1:**
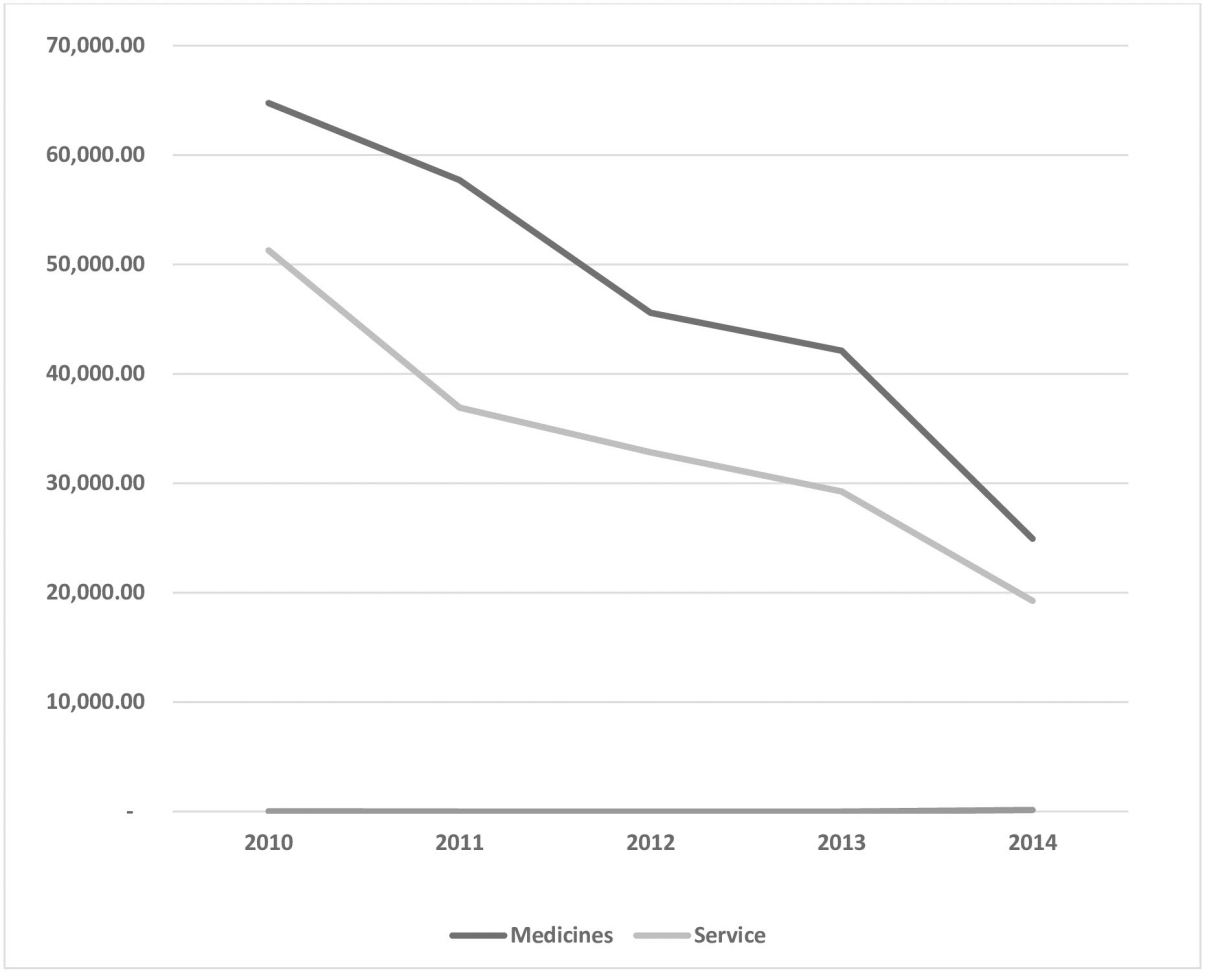
Trend in out-of-pocket payment for healthcare services and medications (2010–2014).

Fig 1 depicts the five-year trend in out-of-pocket payments for medications and services for the five-year period, and evidence of a monotonically decreasing trend in out-of-pocket payment for health service and medication from 2010 to 2014.

**Fig 2:**
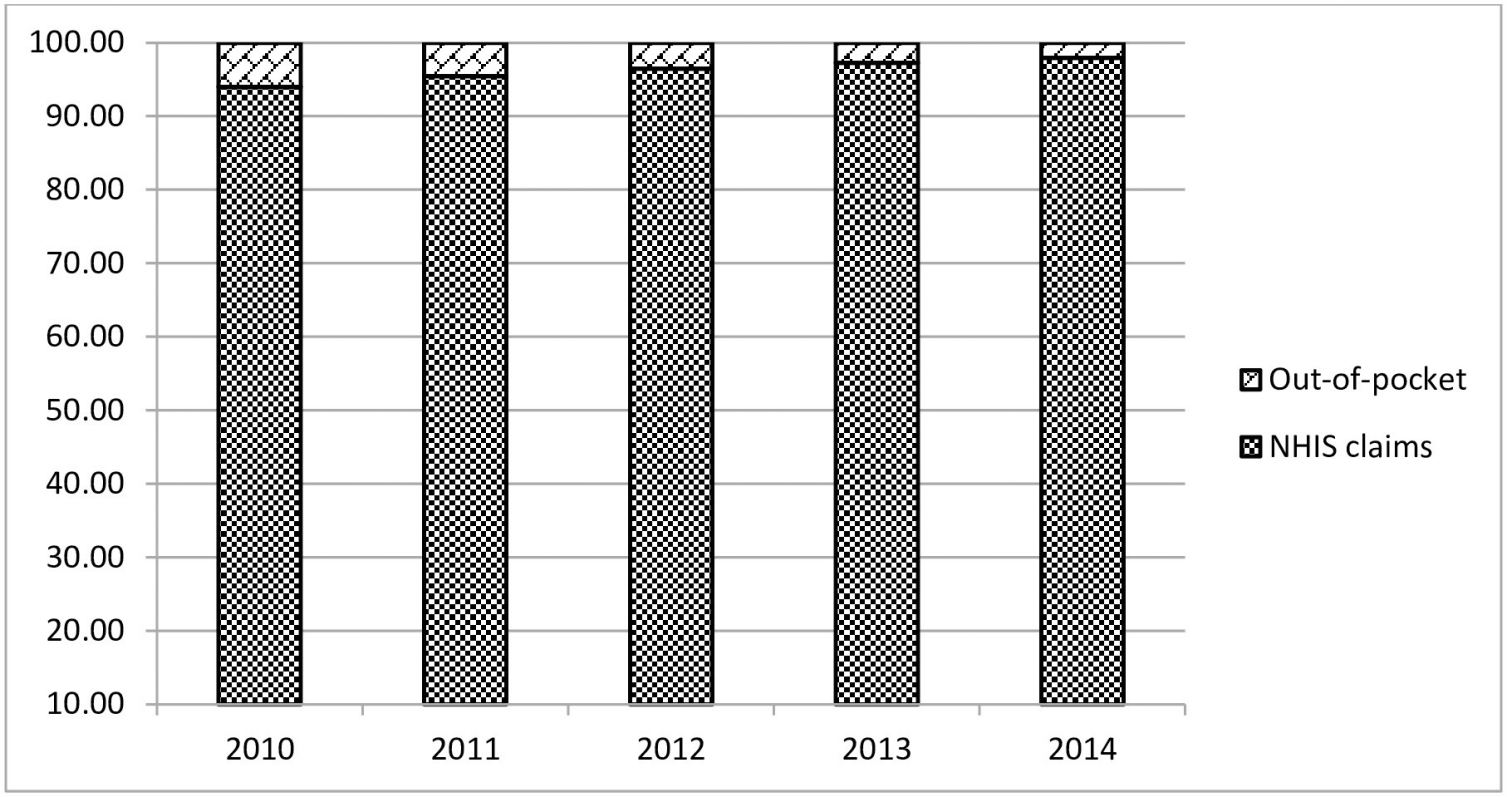
Comparison of out-of-pocket payment and health insurance claims (2010–2014).

Fig 2 is a comparison of the percentage distribution of out-of-pocket healthcare payment and health insurance claims over the study period. In 2010, while insurance claims were 94% of all revenues accruing to primary healthcare facilities in the seven study districts, out-of-pocket payments were only 6.03%. This percentage further dropped to 4.53%, 3.54%, 2.74% and 2.07% for the years 2011, 2012, 2013 and 2014 respectively. Percentage of insurance claims, however, increased steadily to 97.93% of total revenue by the year 2014.

The mean comparison test (t-test) presented in Table 2 shows that the mean reduction in out-of-pocket payment comparing the year 2010 to that of 2012, 2013 and 2014 was significant at 5% level of significance. The mean reduction in health insurance was however only significant when comparing 2010 to 2013.

**Table 2:** Mean comparison test (t-test) using 2010 as comparison year.

Out-of-Pocket Payment				Insurance Claims		
Year	Mean	Std. Err.	Pr (|T| > |t|)	Mean	Std. Err.	Pr(|T| > |t|)
2010	4144.397	302.5217		64609.65	6063.325	
2011	3379.288	424.531	0.148	71272.24	8741.706	0.534
2012	2801.082	315.9346	0.003	76219.43	5394.353	0.158
2013	2548.958	350.6245	0.001	90330.18	8555.829	0.017
2014	1577.975	217.9629	<0.001	74989.96	5404.659	0.207

Results of the generalized estimating equation regression models are presented in Tables 3 and 4. The results reveal that with each progressive year, out-of-pocket payment reduced by $ 319.95 (p-value 0.046). Also, with a unit increase in the number of community-based health service (CHPS) facilities, the level of out-of-pocket payment reduced by $167.58 (p-value 0.011). However, the confidence intervals for these two variables are large which depicts the relatively small nature of our sample size, while this do not necessarily imply that there is no association, it implies that conclusions should be interpreted carefully since a wide range of possible hypotheses exit. Population, number of health centers in a district, the availability of a district hospital and the quantum of insurance claims were not significantly associated with the level out-of-pocket payment.

**Table 3:** Generalized estimation equation model for out-of-pocket payment.

Determinants	Coefficient	Std. Err	P>|z|	[95% Conf. Interval]	
**Year Observed**	-319.95	160.37	0.046	-634.27	-5.64
**Quarter**	29.35	129.53	0.821	-224.53	283.23
**Population(District)**	0.00	0.00	-0.140	-0.01	0.01
**No. CHPS Compounds**	-167.58	66.08	0.011	-297.09	-38.07
**No. Health Centers**	-65.46	83.84	0.435	-229.78	98.86
**Availability of District Hospital**	-603.47	327.11	0.065	-1244.59	37.65
**Insurance Claims**	0.01	0.00	0.143	0.00	0.02
**Constant**	6000.26	946.15	<0.000	4145.84	7854.68

**Table 4:** Generalized estimation equation model for insurance claims.

Determinants	Coefficient	Std Err	P>|z|	[95% Conf. Interval]	
**Year Observed**	12988.72	2567.70	<0.000	7956.12	18021.32
**Quarter Observed**	9524.33	2073.91	<0.000	5459.55	13589.11
**Population(District)**	0.32	0.06	<0.000	0.19	0.44
**No. CHPS Compounds**	-4524.30	1095.58	<0.000	-6671.59	-2377.01
**No. Health Centers**	7240.74	1306.53	<0.000	4679.99	9801.49
**Availability of District Hospital**	2361.91	5681.09	0.678	-8772.82	13496.64
**Out-of-Pocket payments**	2.11	1.44	0.143	-0.72	4.93
**Constant**	-16672.98	18378.74	0.364	-52694.65	19348.69

Table 4 reveals that the year of observation, quarter of observation, population, number of community-based health service (CHPS) facilities and number of health centers were all statistically associated with the level of health insurance claims. With each passing year, insurance claims were found to increase by about $12,988.72. Also, insurance claims increased with the quarter of observation, population and number of health centers in a district. There was however a reduction of about $ 4524.30 with a unit increase in the number of community-based health planning and services (CHPS) facilities.

## Discussion

This paper has analyzed health facility revenue data by segregating them by out-of-pocket health care payments and health insurance claims for primary healthcare over a five-year period. The goal is to contribute to the evidence-base on the contribution of Ghana’s national health insurance scheme on out-of-pocket health payment. Prior to the introduction of the national health insurance scheme in Ghana, the incidence of financial catastrophe and impoverishments due to out-of-pocket healthcare payments in Ghana was relatively high [4, 35]. As of 2005, out of pocket healthcare payments contributed to 9.4% increase in the absolute number of Ghanaians living in poverty using a poverty line of $1.25/day income, and a 31% deepening of the poverty levels of poor individuals in Ghana [4]. Although there is still a significant level of out-of-pocket payments in Ghana [19, 20, 23], our results show that at least at the primary healthcare level in public facilities, out-of-pocket payments are significantly declining alongside the uptake of health insurance. We, however, observe that the rate of increase in insurance claims is lowest in the last year of observation (2014). Although this might suggest a move towards saturation, the reason for this is not clear. Further studies may aim to explore the trend beyond 2014 in order to gain a full understanding of this phenomenon.

Our results are however consistent with other studies showing the financial protective abilities of Ghana’s national health insurance program [21, 36]. For instance, Nguyen et al in their study of two districts within the middle belt of Ghana found that those insured under the national health insurance scheme were significantly less likely to incur catastrophic health care payment [21].

Regression analyses on the factors associated with out-of-pocket and insurance claims show that year of observation and number of CHPS facilities are significantly associated with out-of-pocket payment. It indicates that with each successive year, within the five-year study period, there has been a significant steady reduction in out-of-pocket payments. Also, an increase in the number of CHPS facilities reduces the level of out-of-pocket payment in a given district.

We also found that year of observation, period of the year (quarter), district population, number of community-based primary healthcare facilities and the number of health centers in a district are significantly associated with the level of health insurance claims. The level of health insurance claims was observed to increase progressively over the period. Although district population was associated with insurance claims, a unit increase in population only results in an increase of $0.32. An increase in the number of CHPS facilities results in a reduction in insurance claims by about $4,524 while an increase in the number of health centers results in a rise in insurance claims by $7,240 the reason for this might be the fact that CHPS operations favor preventive healthcare—much of which is intended to be exempt from charges. Thus, expansion of CHPS may be contributing to a reduction in treatment costs overall, compared to reliance on hospitals and health centers which favor curative healthcare services, are more likely to be reimbursable, and could increase overall health costs in the long run.

Results of this study are consistent with those of previous studies that have shown that although out-of-pocket healthcare payments still exist in Ghana, the national health insurance program is having a positive impact on out-of-pocket healthcare payment and indeed has a protective effect on the financial burden of accessing healthcare [19–21]. The implications of the outcome of this study in the light of the existing literature on the impact of Ghana’s insurance program on out-of-pocket payment is that it complements and reinforces the population-based studies that have found insurance to have a financial protective effect on healthcare payments. Although these studies have also argued that substantial out-of-pocket payments still exist [19, 21], Findings of this study show that these payments are not pronounced at least at the primary healthcare level.

### Study limitations

This study used data from public primary healthcare facilities, it is possible that out-of-pocket payment in private health facilities and higher levels of care could be different, the impact of insurance on out-of-pocket payment might, therefore, be overrated by this study. This notwithstanding, this study provides reliable evidence on the contribution of Ghana’s health insurance on out-of-pocket health payment at the primary healthcare level.

## Conclusion

This study has used health facility-based data to contribute to understanding the extent to which Ghana’s health insurance program is contributing to removing financial barriers to primary healthcare delivery in Ghana. The evidence confirms that Ghana’s health insurance program is meaningfully reducing out-of-pocket healthcare payments for primary healthcare in Ghana. There is, therefore, the need for policy makers and implementers to ensure its sustainability. Also, efforts should be made to make it easier for the informal sector workers to fully participate in the insurance program so that Ghana’s move towards universal health coverage can be accelerated.

## Supporting information

S1 Table (DOCX)

S1 Dataset (XLSX)
