# Supplementary material for: Out-of-pocket payment for primary healthcare in the era of national health insurance: Evidence from northern Ghana
Source: PLoS One. 2019 Aug 20;14(8):e0221146. doi: 10.1371/journal.pone.0221146 (PMC6701750; doi:10.1371/journal.pone.0221146)
Supplement: S1 Table — (DOCX) [file pone.0221146.s001.docx]

**Supplementary Tables**

**Table 1: Description of Revenue Categories**

| ***Revenue Category*** | ***Description of Expenditure Inputs*** |
| --- | --- |
| ***Medicines/Drugs*** | This included all revenue accruing from drugs, vaccines, medical consumables and non-medical consumables such as laboratory test kits, plasters, strings etc. |
| ***Service*** | Included revenue accruing from healthcare services such as consultation, worn dressings, administrative services including surgical operations etc. |
| ***Obstetric Care*** | Include all revenue accruing from antenatal and post-natal care services |

**Table 2: Background Characteristics of Study Districts**

| District | Population | Hospital | Health Centers/Clinics | CHPS Compounds |
| --- | --- | --- | --- | --- |
| Garu-Tempani | 136,087 | 0 | 4 | 18 |
| Builsa | 97,343 | 1 | 5 | 21 |
| Bongo | 88,502 | 1 | 6 | 18 |
| Bawku Municipal | 227,983 | 1 | 2 | 13 |
| Bawku West | 98,435 | 1 | 4 | 13 |
| Talensi-Nabdam | 120,403 | 0 | 3 | 12 |
| Bolgatanga M. | 137,706 | 1 | 9 | 11 |
| Total | **906,459** | **5** | **33** | **106** |
